# Finding the loopholes: a cross-sectional qualitative study of systemic barriers to treatment access for women drug court participants

**DOI:** 10.1186/s40352-015-0026-2

**Published:** 2015-06-17

**Authors:** Diane S. Morse, Jennifer Silverstein, Katherine Thomas, Precious Bedel, Catherine Cerulli

**Affiliations:** 1 University of Rochester School of Medicine, Department of Psychiatry, 300 Crittenden Blvd., Rochester, NY 14642 USA; 2 University of Rochester School of Medicine, Women’s Initiative Supporting Health Center for Community Health, 300 Crittenden Blvd., Rochester, NY 14642 USA; 3 University of Rochester School of Medicine, LIVV and Susan B. Anthony Center for Women’s Leadership, 300 Crittenden Blvd., Rochester, NY 14642 USA

**Keywords:** Justice-involved women, Drug treatment court, Social determinants of health, And socio ecological model

## Abstract

**Background:**

Therapeutic diversion courts seek to address justice-involved participants’ underlying problems leading to their legal system involvement, including substance use disorder, psychiatric illness, and intimate partner violence. The courts have not addressed systemic hurdles, which can contribute to a cycle of substance use disorder and recidivism, which in turn hinder health and wellness. The study purpose is to explore the systemic issues faced by women participants in drug treatment court from multiple perspectives to understand how these issues may relate to health and wellness in their lives.

**Methods:**

Qualitative thematic framework analysis of five separate focus groups consisting of female drug treatment court participants, community providers, and court staff (*n* = 25). Themes were mapped across the socio-ecological framework and contextualized according to social determinants of health.

**Results:**

Numerous systemic factors impacted women’s access to treatment. Laws and legal policies (governance) excluded those who could potentially have benefitted from therapeutic court and did not allow consideration of parenting issues. Macroeconomic policies limit housing options for those with convictions. Social policies limited transportation, education, and employment options. Public policies limited healthcare and social protection and ability to access available resources. Culture and societal values, including stigma, limited treatment options.

**Conclusions:**

By understanding the social determinant of health for women in drug treatment court and stakeholder’s perceptions, the legal system can implement public policy to better address the health needs of women drug court participants.

## Background

With the emergence of therapeutic courts, the legal system was transformed as a setting in which defendants can leave their court experiences with more than an incarceration term and bring additional societal benefits. Decreases in emergency room visits, criminal behavior, incarceration, and unemployment led to over a 7:1 cost savings (Ettner et al. 2006). Therapeutic courts seek to address the underlying problems leading to justice involvement, including substance use disorders, psychiatric disorders, and intimate partner violence. The ensuing specialty courts include drug treatment court (DTC), mental health court, veteran court, and human trafficking court.

DTC is designed to help a substance-abusing justice-involved individual link with care and stop abusing drugs (Rossman et al. 2011; Wenzel et al. 2001). To achieve these goals, DTCs engage with community partners that provide services targeting the behavior the legal system hopes to change with the use of “legal leverage.” J. Steven Lamberti, M.D. coined the term “legal leverage” to describe a court process which can restrict freedom in order to improve adherence to a particular treatment plan as an alternative to long-term incarceration (Dentzer 2007). Legal leverage can take many forms including DTC, probation, alcohol monitoring systems and short-term incarceration, which aim to encourage behavior change in the individual to prevent harm to him/herself and the community (Dentzer 2007). This effort to reach out to meet the participant’s needs—wherever they are, is an attempt to break down barriers to care (Lamberti et al. In Press). While these efforts are important steps in diverting DTC participants out of jail and prison and back into the community, there has been a noticeable absence of health partners in these efforts (Wenzel et al. 2001).

DTC has proven though variable effectiveness. DTCs decrease recidivism by an average of 10 % for adults but graduation rates vary widely from 10–90 % (Schaffer 2006). After DTC participation, these individuals are less likely to use substances, engage in criminal behavior, and to need help with education or employment (Rossman et al. 2011). Transitional housing use and substance use disorder treatment are increased, while cost savings are noted. However, no consistent differences are found in employment rate, income, family support, homelessness, and depression symptoms. While those with justice involvement and substance use disorders demonstrate increased health risks (Binswanger et al. 2007) DTC studies have not prioritized or demonstrated an impact on health, generally using self-report of chronic medical problems as the only measure (Binswanger et al. 2007; Morse et al. 2014). This deficiency is rooted in the disconnect between public safety and public health wherein a criminal justice-centered approach takes the place of a public and individual health-centered approach to the health problem of substance use disorder (Drug Policy 2011).

Attempts to assess mechanisms of DTC improvements have indicated varying results including: judges’ support; case management frequency; substance use disorder treatment quality; ethnic, racial, and criminogenic diversity of population served; consistent and prompt legal leverage including individualized rewards; and post-treatment services. Perhaps most importantly, assessments and reassessments are crucial and underutilized (84–97 %) in order to allow for data-driven decisions on needs and substance use disorder treatments delivered. Policy recommendations include continued DTC funding with use, development, and more research on the impact of evidence-based practices (Rossman et al. 2011; Taxman 2014; Schaffer 2006). Building a bridge between DTCs and health care providers is recommended in order to best meet participant and community needs, but is not advanced by national DTC leaders (Wenzel et al. 2001).

DTC national standards include gender and culturally-specific practices with improved outcomes when they are practiced, but must be delivered by trained and supervised individuals using evidence-based practices (Adult drug court best practice standards 2013). Recommended treatment addresses trauma and psychiatric histories (Schaffer 2006). However, DTC studies to date have not successfully studied the treatment actually delivered (Schaffer 2006). Because less than half of evaluated programs address known criminogenic factors such as education, parenting, employment, and peer contacts (Schaffer 2006) it is recommended to evaluate and address these social needs (Adult drug court best practice standards 2013). Other recommendations include establishing a “participant profile” which includes substance use disorder problem severity and need for supplemental services. Furthermore, getting participant and case manager feedback is advised to help measure DTC success (Rempel 2005; Schaffer 2006). The extent to which these legal, social, and treatment recommendations are implemented is not known but are inconsistent across DTC courts in the United States (Rempel 2005; Schaffer 2006) and do not address systemic health barriers or impacts.

The Social Determinants of Health (SDH) Framework can help to elucidate challenges that justice-involved individuals face. It complements the “Eco-Social Model” (Altice 2013) of the health and justice framework, expanding the systemic context. Social determinants of health are social, physical and economic environments or ecosystems, which create social hierarchies and impact health outcomes, including mortality rates. (Marmot et al. 1978, 1991). For women DTC participants, it is important to better understand legal and social policy barriers to employment, education, housing, and transportation which may critically impact their functioning in the community and health outcomes. DTC stakeholder (women, community providers and court staff) perceptions on the impact that DTC policies has on these determinants are also unclear.

The aim of this study is to explore the systemic issues faced by women DTC participants from multiple perspectives in order to understand how these issues may relate to their health and wellness. Additionally, the study aims to discover where public policy can address gaps in health and improve the social conditions not conducive to successful re-entry from DTC and incarceration.

## Methods

### Study design

We conducted five stakeholder focus groups in 2012: two with women DTC participants, two with court staff, and one with providers from two community agencies chosen because they serve women in DTC. One of the community agencies serves medical and social needs of HIV patients and the other one serves survivors of intimate partner violence (IPV) (Kitzinger 1995). Recruitment was conducted through three primary methods: 1) Approaching women outside a county DTC in a moderately large upstate city in the state of New York; 2) Emailing via staff listserves; and 3) Snowball methods of staff contacting other staff who gave the team permission to email them. There were 82 women who participated in DTC during the recruitment year out of a total of 320 DTC participants. Court staff were recruited from the DTC judge, 20 case managers, two public defenders who serve DTC participants, one district attorney who works with the DTC population, and two DTC court clerks. The agencies serving HIV patients and IPV survivors each has over 50 staff members, however focus group participants were recruited from those designated by directors to be knowledgeable about justice-involved women. Recruitment scripts were used for approaching women and emailing staff. Focus groups were in private conference rooms as follows: a community health center (DTC participants), an HIV/AIDS clinic (providers), and in court (court staff). Focus groups were audio-recorded, transcribed and de-identified. Lines within transcripts were labeled to differentiate between focus group facilitators and participants. Focus groups subjects were provided a meal and reimbursed with $10 gift cards. Court employees were required by employers to decline reimbursement.

Research team members developed the focus group guide according to research goals, focus group methodology (Morgan and Spanish 1984), and principles of community based participatory research (Israel et al. 2010; Sormanti et al. 2001). The guide (see Table 1 for sample questions. Full guide available upon request) was modified slightly according to focus group member roles (DTC participant, providers, or court staff) and revised iteratively over the course of the study. Questions addressed personal and systemic barriers and facilitators to accessing healthcare. Two experienced focus group facilitators/co-investigators, followed the discussion guide, attended to group responses, and allowed a spontaneous exchange of ideas (Brown 1999). The university Institutional Review Board approved the protocol as low risk so in lieu of consent, participants were provided with an information sheet. Participants completed anonymous demographic information sheets (see Table 2).

**Table 1:** Focus group discussion guide for community and court staff

What problems do you think the DTC participants face accessing health care services?	
- How severe do you think those problems are?	
What about your coworkers? What do you know about their experiences with the DTC or similar participants?	
Could you describe your experiences with DTC or similar participants?	
Were you able to help your DTC or similar participants?	
If yes, how?	
If not, why?	
How many DTC or similar participants do you encounter in a day?	
What can we do to increase access of health service providers for the DTC participants?	
How often do you refer your DTC or similar participants to other health care services?	
What barriers do you think the DTC participants face when searching for health services?	
How should we address these barriers?	
What do you think can motivate women in DTC into searching and receiving health services?

**Table 2:** Focus group demographics

Source	N	Age	Sex	Race	Ethnicity	Education
Drug treatment court	*N* = 7	39.9 ± 12.9	F-7	C-5, AA-2	—	<High School-some college
AIDS and IPV providers	*N* = 9	45.7 ± 7.1	F-7	C-4. AA-5	H/L-2	GED-Graduate degree
Court staff	*N* = 8	42.8 ± 8.6	F-5	C-8	—	Graduate degree

F- Female, C- Caucasian, AA- African American, H/L- Hispanic/Latino

### Data analysis

A multidisciplinary analytic team (which included personal experience as a DTC participant, an attorney, a physician, and trained graduate and undergraduate research assistants), identified recurrent events, terms, and social actors within each transcript. We then organized recurrences into higher order conceptual themes (Creswell 2012). The research team conducted the analysis in several large group consensus meetings, which included 4 undergraduate and graduate research assistants. The team first coded each focus group transcript in pairs or triads which were then brought to large group meetings and analyzed in detail by consensus. The team utlized ATLAS.ti software to code the data (Smit 2002) and enter them into the ATLAS.ti system. The lead author reviewed the codes and reached consensus with the team. The team used the framework approach (Pope et al. 2000) in which investigators by consensus identify a model (or framework) relevant to the data and map the data according to the model. The team identified in this data set, themes across the socioecological model, which included intrapersonal, interpersonal, institutional, community, and systemic barriers and facilitators to healthcare. In the analysis for this manuscript, we further identified a framework for the systemic barriers and facilitators as the World Health Organization (WHO) Social Determinants of Health model (Solar and Irwin 2010). Team members then selected those themes and quotes categorized as system-related and categorized them according to the Social Determinants of Health model, with the rest of the team agreeing by consensus. We sought representative quotes, including similarities and differences between participants’ views, and diverse representation among healthcare providers, court staff, and women in DTC.

The framework analysis method determined the categories which addressed the socioeconomic and political context and its relationship with individual determinants of health according to the Social Determinants of Health model. Final coded quotes and themes were agreed to by the research team and tabulated (Table 3). A key community informant reviewed the data and conceptual framework as respondent verification (Barbour 2001).

**Table 3:** Numbers and percentages of quotes from focus groups relating to the social determinants of health (SDH) framework for socioeconomic and political context

Quote distribution according to SDH model	n	%
Governance	26	13.2
Macroeconomic policies	54	27.4
Social policies	23	11.7
Public policies	58	29.4
Cultural and societal values	36	18.3

## Results

### Framework

Using the social determinants of health framework, we focused on the *socioeconomic and political context* for social inequities which lead to health inequities: governance, macroeconomic policies, social policies, public policies, and culture and societal values.

### Governance (laws and legal policies)

Participants described how laws governing eligibility for diversion court, convictions, and sentencing impact their health in unexpected ways when they are not offered DTC or lack incentive due to the nature of their charges:

“…A lot of our female clients are charged with prostitution and…have combined chemical dependency and mental health issues… [which have] failed to be addressed in the past… A lot of them don’t qualify for drug court. [They] can’t get into mental health court because prostitution is a misdemeanor, not a felony, so there’s no real appropriate avenue to send them.” (Court Staff)

Participants also described laws that govern DTC policies and prevent women’s specific needs as mothers from being taken into account:

“If your son is sick…you can’t [miss court]…I can leave him home and he’s 13, but he has problems. I had to leave him [when] he was sick…Thursday. I had to go to mental health and group and he stayed home without anyone.” (Woman in DTC)

### Macroeconomic policies

Because of their convictions, the women were not eligible for housing loans or numerous rentals. Lack of safe affordable housing contributed to many problems.

“They can't get out of that neighborhood…because when they apply for a house and they do that background check and all that they're stuck…They're limited to where they can move.”

(Community Provider)

### Social policies

Criminal history was described as a barrier to obtaining employment (and health insurance) and when superimposed on transportation, made it hard to get healthcare:

“You might want to go to the doctors but if you don't have insurance or you don't have transportation [it is very difficult]. [If] you want to get a job but you have a prior criminal history [it is very difficult] and it's hard to get into work.” (Community Provider)

Transportation was a frequently described barrier in all focus groups, including a lack of knowledge among women, their providers, and court staff as to exactly what were the available transportation resources:

“It’s hard to go to mental health if you have even a little one with you…not having a ride, providing bus passes, providing you know [about them]…because. I have 150 dollars a month that I’m supposed to support two children and myself on right now.” (Woman in DTC)

“If you live on the west side and your treatment provider is over at Hospital 1 trying to get there… you have to leave at 6:30 in the morning to get to a 2:00 appointment because you have to get the transfers and you have to make sure that you get on that bus, and you have to then be able to a walk a certain amount.” (Court Staff)

There was concern about a cycle of incarceration, unemployment, lack of housing, and relapse:

“When you get out…finding employment and whatever you may apply for you have to always list your criminal history or convictions and that is such a big barrier to housing and a lot of other programs, especially when it comes to employment. As soon as you say you have a conviction you're automatically out the door…that a lot of times puts people in a position [to ask]… ‘Where am I making any progress? …I'm on parole, I have a drug history so as soon as I go in…’ Whether or not they have an education from college or just have training or skills that automatically puts [them] at a disadvantage. So [they're] gonna get denied when it comes to housing, to a job…There needs to be something put in place where these women are not always rejected because that can cause them to go back to using….It's a big problem, a very big problem.” (Court Staff)

### Public policies

Multiple groups decried delays in psychiatric treatment as a contributor to relapse. When a long wait for psychiatric treatment and her psychiatric medications ended in a relapse and subsequent incarceration, one participant lost her job, which caused more stress:

“Now that I’m taking the medication I’m not feeling [like] drinking or using but now I really have issues with dealing with this job loss.” (Woman in DTC)

Another reason for missed healthcare was financial and lapses in health insurance:

“A lot of my clients can’t afford a doctor’s appointment even if it’s a sliding scale because of their low income and they don’t have the proper benefits in place to cover that.”(Court Staff)

Combining substance use disorder treatment with housing for themselves and their children helped participants to focus on needed health and recovery:

“When they go to residential they know they have a warm bed, they know they’re gonna be fed three meals a day, they know they have a place to come home to, so it makes them more able to focus on their health…contraceptive services…going to the dentist….All of those things because they’re not worrying about where am I gonna sleep tonight, what am I gonna eat, is somebody gonna take my kids away because we’re sleeping on a bench?” (Court Staff)

Lack of education, health literacy, and complexity of the healthcare system was noted as a barrier to health:

“Part of it comes down to education. Because even those of us that each and every day work with the system…have many of these issues. Even we aren’t able to keep up with every single thing that’s going on.” (Court Staff)

“All the medications…and this is another thing with these meds…you wanta take one for something but then it’s gonna give you this. Wait a minute…isn’t that what I’m taking it for?” (Woman in DTC)

Adding to the complexity of attaining sobriety, housing, and health is seeking physical safety from an abusive partner:

“When you’re in a domestic violence situation…she’s moving from place to place to place to place to place to place so that this person doesn’t find her.” (Court Staff)

### Culture and societal values

There can be a variety of reasons that stigma prevents women from getting substance use disorder treatment:

“Specifically for white, middle-class women. [there is] embarrassment over the diagnosis. Embarrassment ‘I got a DWI. I can’t drive anymore. I have to take a bus. It’s all beneath me.’”(Court Staff)

Providers were frustrated with client mistrust, suspected racial bias, and not seeing anybenefit of urine drug testing when it was done by community medical providers who do not have the authority to incarcerate based upon the results. This provider saw potential advantages:

“They try to say…because this person isn't my color they don't understand where I'm coming from or they don't care about me, not looking at the whole bigger picture of what they're trying to do…with the urine [toxicology] tests for example; they don't see that it's about the drug interactions and [their] health. They're seeing it's the provider who is white telling them what to do.” (Community Provider)

Other community providers expressed understanding of how stigma could prevent getting treatment:

“Clients have experienced…people treating them in a [negative] way…It's to be expected…You use drugs…You're lower income…Your children have these other issues…Sometimes…some of that stigma and shame do come in. [They] don't want to admit that [they’re] dealing with this cause [they] don’t wanta be another statistic or feed into…some of these stereotypes that people already believe about [their] community or group.” (Community Provider)

## Discussion

In this analysis, findings revealed women face complex systemic barriers to attaining health and wellness when participating in DTCs. These systemic barriers are viewed within the framework of the Social Determinants of Health, which includes: *governance (laws and legal policies), macroeconomic policies, social policies, public policies, and culture and societal values* (Solar and Irwin 2010)*.* Participants described specific systemic inequities impacting the health and wellness of women in DTC, including lack of affordable and safe housing, education, limited employment opportunities due to criminal history, inadequate transportation, and insufficient healthcare access upon release from incarceration. While much of the effort involving DTC involves overcoming individual-determinants of health, preventing and eliminating these barriers at systemic levels has the potential to increase participants’ success. DTC policies seek to balance defendants’ accountability with addressing underlying problems, but the courts have not taken the opportunities to address systemic hurdles and social determinants which can contribute to a cycle of substance use disorder and recidivism, simultaneously hindering health and wellness (Solar and Irwin 2010; Wenzel et al. 2001, 2004). We find that inconsistent application of expert recommendations and a lack of clear focus have perpetuated the systemic, psychiatric, and physical health barriers that may prevent a defendant from engaging in the court process. The obvious healthcare partners for treatment courts include substance use disorder providers and psychiatric providers. However, a much larger range of service and community providers could be engaged to have greater success in meeting the myriad needs of DTC participants. Insuring wrap-around services for women in DTC who have synergistic risks of substance abuse, violence, psychiatric disorders, inadequately treated medical problems, and HIV/AIDS can be a first step (Meyer, Springer, and Altice 2011).

The governance of drug court is established by state and local governing bodies (NYCourts.gov 2014). One concern expressed was that DTC eligibility requirements limit participation of women in DTC, particularly women arrested on prostitution charges. While the eligibility requirements of DTC vary even within states, it is critical to assess if program requirements are restricting individuals that would benefit from participation by explicitly excluding those with dual diagnoses (substance use and psychiatric disorders) from DTC participation (Evans, Li, and Hser 2009). Increasing numbers of human trafficking courts may help bridge these gaps. Additionally, focus group participants address the need for individualization of protocol and policies to encourage being a successful DTC participant and parent. Some studies have indicated that having children helps justice-involved women to stay sober; court staff could build upon this motivator and adjust requirements when women are caught up with their children’s needs. (Pelissier and McCarthy 1992).

Although discrimination based upon history of incarceration is illegal, focus group participants identified criminal justice involvement as a barrier to education, employment, and housing. Many employers, universities, and colleges ask the applicant to check a box if s/he has a history of incarceration, despite the recent increase in ‘Ban the Box’ laws throughout the country (National Employment Law Project 2014). Incarceration history can dramatically limit an individual’s ability to find employment (Morris et al. 2008; Harris and Keller 2005). In terms of education, policies limiting student loans for higher education may prevent attaining a degree, even if admitted despite their criminal background (United States Department of Education 2010). Focus group participants noted the double bind of women who did not qualify for the support of a diversion court because they were convicted of non-felony prostitution while conversely those with a felony conviction suffering from a subsequent lack of education, housing, and employment.

Adequate transportation and safe housing were identified as important resources to achieving DTC goals as well as health. Nonetheless, the US Department of Housing and Urban Development under The Quality Housing and Work Responsibilities Act (QHWRA) allows property owners and management the authority for screening and denial of federally-assisted housing program to individual and families with specific types of criminal activities or history (Hunt et al. 1998). These social policy barriers challenge an individual’s ability to participate in substance use disorder treatment as well as access healthcare needs. The World Health Organization suggests that the socioeconomic and political determinants of health are not adequately addressed in policies and protocols (Solar and Irwin 2010). Individual effects based upon such policies might include a DTC mandate that a participant secure safe housing, but in that community, there is a severe shortage of housing for individuals who have criminal histories. The US Department of Housing and Urban Development (HUD), regulates housing for low-income families and encourages public housing authorities to take into consideration factors that mitigate criminogenic risk (Federal Interagency Reentry Council). However local authorities have the final say and may deny access unfairly.

Stigma and shame surrounding substance use disorder were also illuminated by the focus group participants. Differences in race, socioeconomic status, sexual identity, or preference between DTC participants and their providers can lead to distrust and disengagement in services. (New York State Office for the Prevention of Domestic Violence 2014). The social stigma of substance use disorder can negatively impact DTC participants’ ability to seek help to improve their health (Oser, Knudsen, Staton-Tindall, and Leukefeld 2009). Similarly, those who are middle class, upper class, immigrants, religious minorities, or hearing impaired can face similar barriers within their own neighborhoods and institutions (Family Violence Prevention 2009; Anderson 2014).

The social determinant domains, taken in their totality, present a complex web of systems that women must navigate. When basic needs for housing, employment, insurance, transportation, and safety are not met (Freudenberg et al. 2005), it is difficult to improve in the way required for DTC, health, and life success. Despite sporadic innovative programming in some DTCs, it is unclear how to get to the broad implementation phase of strategies which are recommended by DTC professionals but interdisciplinary approaches may be helpful (Adult drug court best practice standards 2013 The lack of this broad implementation may be a factor in conflicting data on DTC effectiveness in general and on health outcomes in particular (Drug Policy 2011). This paper adds to the literature by examining how the social determinants of health are experienced by women in DTC through exploring the varying perceptions of the women, community providers, and court staff regarding the interface with DTC and these systemic inequities. Community based participatory research strategies help to inform novel strategies (El-Bassel et al. 2012; Israel et al. 2010). These perceptions can inform how drug court policies might improve DTC participants’ physical and psychiatric health while also reducing recidivism. Specifically, we focus on female drug court participants’ experiences in a specialized city court. It is important to address the needs of this particular population for many reasons. Women involved in the justice system report more health problems than other women (Staton et al. 2003). DTC participants report significantly greater prevalence of psychiatric illness than the general population (Goff et al. 2007; Resnick et al. 1993). These women are also at greater risk for sexual and physical assault and consequent sexually transmitted infections (El-Bassel et al. 2012). Given DTC participants' co-morbid physical and psychiatric health needs, and the unique issues female drug users face, this paper explores how systemic issues affect access to a wide variety of resources which play a role in one’s health.

This exploratory pilot research has limitations but adds to the knowledge of a unique and understudied population of women DTC participants. Our sample had limited racial and ethnic diversity (only two Hispanic participants overall and no people of color among court staff), which may have decreased discussion of the systemic issues of racial and ethnic discrimination. This study was conducted in a single geographical location which may limit examples of systemic barriers. Nonetheless, findings were reinforced with respondent verification, a member of the research team with experience as a DTC participant, and a member of the team with experience as both a district attorney and a public defender.

## Conclusions

This study begins to close the gap in examining how DTC policies and procedures might go further in promoting women’s health. It is important for courts to understand how their policies may directly impact these women’s daily challenges, both positively and negatively, to navigate health. To address this issue, political leadership is essential. An example of a potential policy change includes funding for drug courts to begin providing training and community linkage for higher paying jobs. Another example would be to advocate for safe housing for those in DTC as a change in public housing policies. This in turn may elevate status of living and community leadership.

**Fig. 1:**
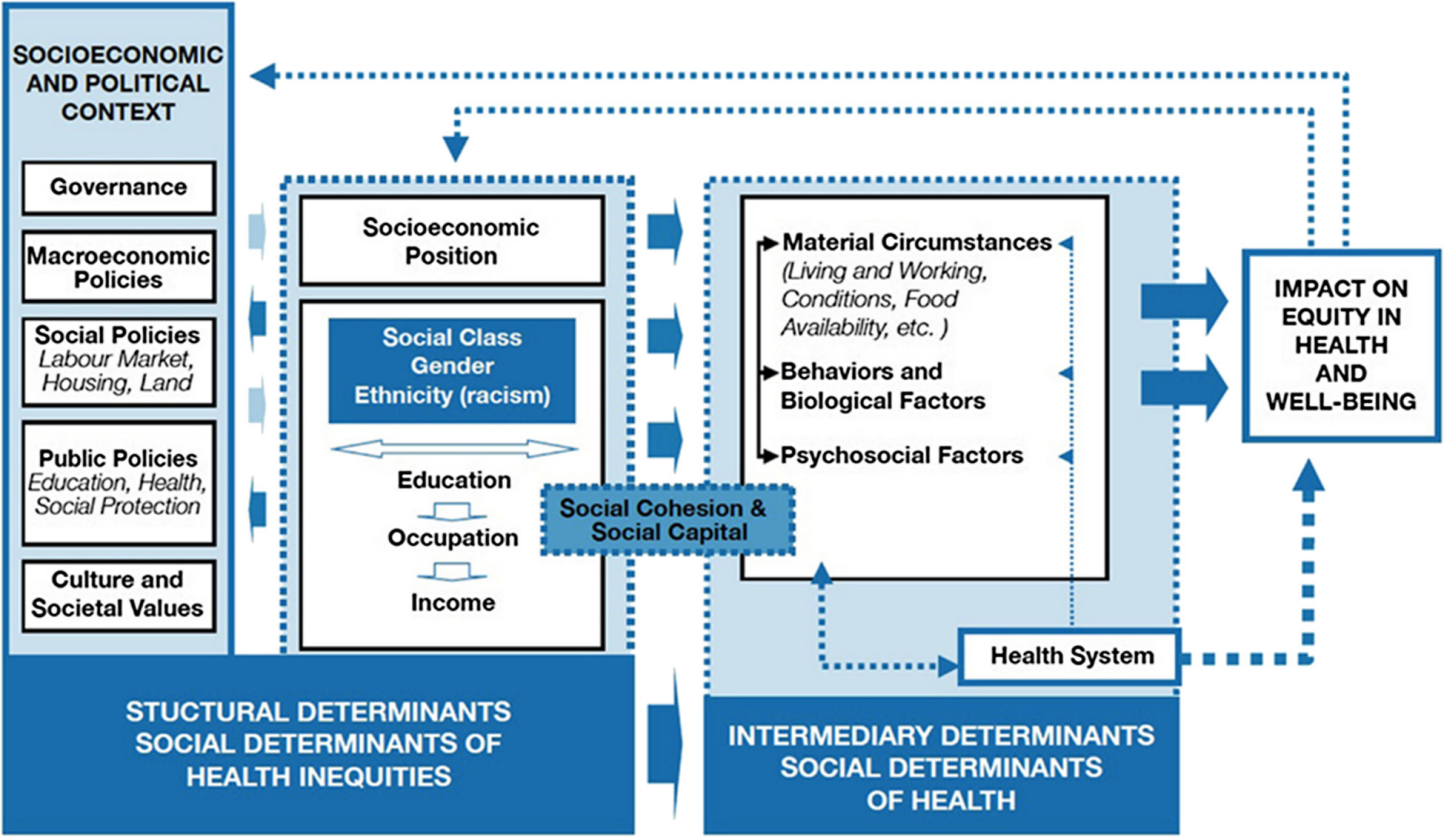
Social determinants of health framework

Currently, courts do not routinely include a feedback mechanism to evaluate the impact of the structural determinants of health and the healthcare system (Fig. 1, Solar and Irwin 2010). The results of this analysis suggest further research is needed to determine whether therapeutic courts could increase health and overcome systemic barriers if they are applied supportively. By understanding the social determinants of health for women in DTC coupled with stakeholder perceptions of DTC, the legal system and policy makers can better address the health needs of women in DTC.
